# The Anastomotic Angle of Hemodialysis Arteriovenous Fistula Is Associated With Flow Disturbance at the Venous Stenosis Location on Angiography

**DOI:** 10.3389/fbioe.2020.00846

**Published:** 2020-07-23

**Authors:** Chih-Yu Yang, Ming-Chia Li, Chien-Wen Lan, Wang-Jiun Lee, Chen-Ju Lee, Cheng-Hsueh Wu, Jing-Min Tang, Yang-Yao Niu, Yao-Ping Lin, Yan-Ting Shiu, Alfred K. Cheung, Yan-Hwa Wu Lee, Oscar Kuang-Sheng Lee, Shu Chien, Der-Cherng Tarng

**Affiliations:** ^1^Institute of Clinical Medicine, School of Medicine, National Yang-Ming University, Taipei, Taiwan; ^2^Division of Nephrology, Department of Medicine, Taipei Veterans General Hospital, Taipei, Taiwan; ^3^Stem Cell Research Center, National Yang-Ming University, Taipei, Taiwan; ^4^Center for Intelligent Drug Systems and Smart Bio-devices (IDS^2^B), Hsinchu, Taiwan; ^5^Department of Biological Science and Technology, College of Biological Science and Technology, National Chiao Tung University, Hsinchu, Taiwan; ^6^Division of Cardiology, Department of Medicine, Taipei Veterans General Hospital, Taipei, Taiwan; ^7^Department of Aerospace Engineering, Tamkang University, New Taipei City, Taiwan; ^8^Division of Nephrology and Hypertension, University of Utah School of Medicine, Salt Lake City, UT, United States; ^9^Veterans Affairs Medical Center, Salt Lake City, UT, United States; ^10^Institute of Engineering in Medicine, University of California, San Diego, San Diego, CA, United States; ^11^Department and Institute of Physiology, School of Medicine, National Yang-Ming University, Taipei, Taiwan

**Keywords:** arteriovenous fistula, angiography, anastomotic angle, stenosis, computational fluid dynamics, disturbed flow

## Abstract

The juxta-anastomotic stenosis of an arteriovenous fistula (AVF) is a significant clinical problem in hemodialysis patients with no effective treatment. Previous studies of AV anastomotic angles on hemodynamics and vascular wall injury were based on computational fluid dynamics (CFD) simulations using standardized AVF geometry, not the real-world patient images. The present study is the first CFD study to use angiographic images with patient-specific outcome information, i.e., the exact location of the AVF stenotic lesion. We conducted the CFD analysis utilizing patient-specific AVF geometric models to investigate hemodynamic parameters at different locations of an AVF, and the association between hemodynamic parameters and the anastomotic angle, particularly at the stenotic location. We analyzed 27 patients who used radio-cephalic AVF for hemodialysis and received an angiographic examination for juxta-anastomotic stenosis. The three-dimensional geometrical model of each patient’s AVF was built using the angiographic images, in which the shape and the anastomotic angle of the AVF were depicted. CFD simulations of AVF hemodynamics were conducted to obtain blood flow parameters at different locations of an AVF. We found that at the location of the stenotic lesion, the AV angle was significantly correlated with access flow disturbance (*r* = 0.739; *p* < 0.001) and flow velocity (*r* = 0.563; *p* = 0.002). Furthermore, the receiver operating characteristic (ROC) curve analysis revealed that the AV angle determines the lesion’s flow disturbance with a high area under the curve value of 0.878. The ROC analysis also identified a cut-off value of the AV angle as 46.5°, above or below which the access flow disturbance was significantly different. By applying CFD analysis to real-world patient images, the present study provides evidence that an anastomotic angle wider than 46.5° might lead to disturbed flow generation, demonstrating a reference angle to adopt during the anastomosis surgery.

## Introduction

An arteriovenous fistula (AVF) is created through anastomosing an artery and a superficial vein of the upper limb in hemodialysis patients to establish an extracorporeal circulation for hemodialysis therapy. Such artificially created fistula alters the blood flow and hemodynamics of the superficial vein, exposing venous endothelial cells to a supra-physiological shear force (Remuzzi et al., 2003). Although the AVF is the preferred access as compared to AV grafts and catheters due to a higher patency rate, its 1-year primary patency rate remains unsatisfactory, ranging from 34 to 56% (Leapman et al., 1996; Hodges et al., 1997; Turmel-Rodrigues et al., 2000; Gibson et al., 2001; Clark et al., 2007; Huijbregts et al., 2008; Li et al., 2008). Therefore, it is crucial to solving the complex pathogenesis of AVF stenosis that is a critical contributing factor of AVF failure (Mezzano et al., 2001; Sho et al., 2002; Paszkowiak and Dardik, 2003; Remuzzi et al., 2003; Asif et al., 2006; Roy-Chaudhury et al., 2006; Lee and Roy-Chaudhury, 2009; Osgood et al., 2014; Yang et al., 2014). The radio-cephalic AVF at the wrist is the recommended first choice for hemodialysis access (Lok et al., 2020). In radio-cephalic AVFs, the juxta-anastomotic region is defined to be around 2–5 cm distal to the AV anastomotic site, and it is the most common location that stenosis occurs in the venous segments (Rajan et al., 2004; Asif et al., 2005; Nassar et al., 2006; Robbin et al., 2018). Usually, a radio-cephalic AVF is created by connecting the artery side to the venous end. The radial artery deviation and reimplantation (RADAR) surgical technique anastomoses the radial artery end and the cephalic venous side to minimize the vasa venorum injury and to counter the restenosis seen in the juxta-anastomotic vein, but this surgery is contraindicated in many patients and is not widely used (Sadaghianloo et al., 2016).

Computational fluid dynamics (CFD) is a powerful engineering tool that helps to elucidate the hemodynamic insult on the vascular wall (Sivanesan et al., 1999b; Van Canneyt et al., 2010; Shar et al., 2020). Once AVF is created, the blood flow in the vein suddenly increases, leading to an increased Reynolds number with its flow pattern converting from laminar to a disturbed one (Ene-Iordache and Remuzzi, 2012; Lu et al., 2014). The venous endothelial cells in AVFs are susceptible to pathological shear stress that is known to induce intimal hyperplasia (Chien, 2007; Chiu and Chien, 2011). We hypothesized that the shape, particularly the anastomotic angle of a radio-cephalic AVF, determined the hemodynamics in the AVF, producing an extreme shear injury at a particular vascular wall location. We tested this hypothesis by using a statistical association.

We took advantage of CFD simulations to analyze hemodynamics in AVFs with various patient-specific shapes and anastomotic angles acquired from individual angiographic images. Because the arterial flow of upper arms and forearms have a different arterial flow, we focused on forearm AVFs to control the arterial flow variable. In the present study, we investigated the radio-cephalic AVF at the wrist. Specifically, we analyzed the relationship between the AV anastomosis angle and hemodynamic parameters (flow velocity, wall shear stress (WSS), and flow disturbance). We also aimed to identify an optimal anastomotic angle of AVF, which might serve as a reference range for vascular surgeons to minimize disturbed flow generation and the subsequent intimal hyperplasia.

## Materials and Methods

### Study Participants

From January 2017 to December 2017, we conducted a cohort study at the Taipei Veterans General Hospital, a tertiary-care referral hospital. This study was approved by the Institutional Review Board of the Taipei Veterans General Hospital. All patients were at least 20 years of age, had end-stage renal disease, had been on maintenance hemodialysis therapy thrice weekly for at least 3 months, and were using a mature radio-cephalic AVF as their vascular access for long-term hemodialysis. A total of 27 hemodialysis patients who received percutaneous transluminal angioplasty (PTA) for juxta-anastomotic stenosis on their radio-cephalic AVF were enrolled. Before the angiography, the presence of stenosis was either detected by ultrasonography or reported with cannulation difficulties by the dialysis staff who is responsible for the cannulation for hemodialysis. The demographic and clinical characteristics, including age, gender, duration of dialysis, the time interval between AVF creation surgery and PTA, underlying renal diseases, comorbidities, and angiographic features, were obtained.

The Institutional Review Board of the institute approved all protocols before the study began, and the protocols conformed to the ethical guidelines of the *Helsinki* declaration. Because of the retrospective nature of the study, the need for informed consent was waived.

### Angiographic Procedure for AVF

By using the digital subtraction technique and radiopaque contrast injection, the angiographic images were obtained to evaluate AVF contour. A lesion was considered stenotic if there was a luminal narrowing greater than 50% of the vascular internal diameter, in which the PTA was performed using an appropriately sized non-compliant balloon catheter advanced over the wire through a corresponding sheath, using a hand syringe assembly. After interventional angioplasty, balloon catheters and sheaths were withdrawn, and hemostasis was obtained with manual compression. The radiocontrast presented on the angiographic image denotes the lumen of AVF. The percentage of stenosis was quantified by calculating the difference between the internal diameter of the upstream non-stenotic vascular segment (d_0_) and the internal diameter of the stenotic lesion site (d_1_). The stenotic ratio was defined as (d_0_ - d_1_)/(d_0_) × 100%.

### Geometric Models: AVF Lumen Reconstruction

**FIGURE 1:**
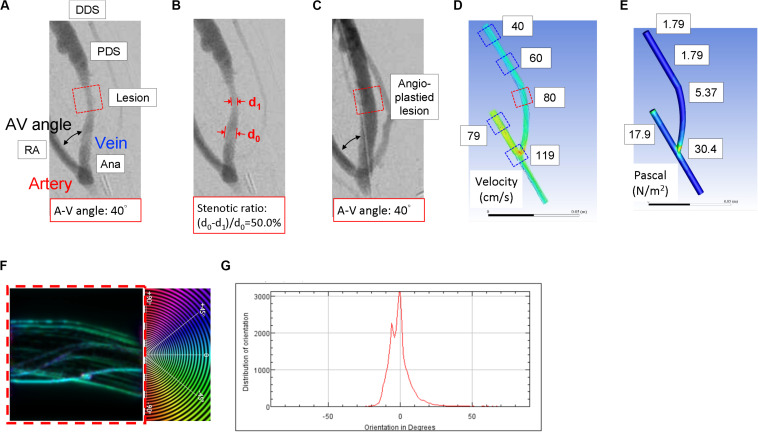
The representative computational fluid dynamics simulation analysis of the blood flow characteristics in the patient-specific 3D-reconstructed arteriovenous fistula (AVF) model. **(A)** A representative angiographic AVF image of the hemodialysis patient before the angioplasty. **(B)** The radiocontrast presented on the angiographic image denotes the lumen of AVF. The percentage of stenosis was quantified by calculating the difference between the internal diameter of the upstream non-stenotic vascular segment (d_0_) and the internal diameter of the stenotic lesion site (d_1_). **(C)** A representative angiographic AVF image after angioplasty for the stenotic lesion. Various features, including blood flow velocity **(D)** and wall shear stress **(E)**, were obtained at five different locations of the 3D geometric model mesh. The red square in the dotted line in **(A)** denotes the stenotic lesion site, one of the five locations of AVF. Numbers in white squares in **(D,E)** are the velocity and shear stress values for the five locations in **(A)**. The stenotic region in **(D)** (red square in dotted line) was analyzed for vectors of blood flow trajectories **(F)** and the distribution plot of blood flow vectors **(G)**. Zero-degree was set to be parallel with the axial direction of the blood vessel; the angle of deviation defined the blood flow vector, and the degree of the most abundant vectors was denoted as the blood flow disturbance. This was also performed for all the five locations of AVF. RA, radial artery; Ana, anastomosis; PDS, proximal downstream site; DDS, distal downstream site.

As shown in Figure 1C, according to the shape and anastomotic angle of two-dimensional (2D) angiography for each patient, a patient-specific three-dimensional (3D) geometrical model of AVF venous lumen was reconstructed by using Autodesk Fusion 360. By using the non-stenotic angiographic image to reproduce the original lumen while the AVF was not stenosed yet, we were able to simulate the process of stenotic lesion development. AVF lumen segmentation was performed to generate a 3D surface in STL (stereolithography) format and was then discretized by using a commercial CFD software (Fluent 18.0, ANSYS Inc., Canonsburg, PA, United States) to obtain a high-resolution mesh, with approximately 20,000–50,000 tetrahedral and hexahedral cells. The first step of reconstruction was to make sure that the images of the blood vessels are in actual size. The second step was to mark out the path of the vessels. We used the curve tool in ANSYS SpaceClaim to trace out the paths of blood vessels. The curves must be at the center of each vessel image. The third step was to extract the features of vessel images. We reconstructed circular sections that are perpendicular to the curve paths. The diameters of circular sections are in consent with diameters of vessels. Note that we drafted a circular section when a change in vessel diameter occurs. The final step was to connect all the circular cross-sections to form a single geometry. Using the connect tool in ANSYS SpaceClaim, we merged circular sections based on paths we previously drew.

### Geometric Models: Meshing Geometries

The first step of meshing was to load our geometry into the meshing toolbox. The second step was to identify the inlet, outlets, and vessel walls. After defining these sections, we used the inflation tool in ANSYS Mesh to mesh the geometry. The reason for using inflation is that inflation created layers of hexahedral cells near the inner part of the walls. Hexahedral cells tend to perform better when predicting wall shear stress. After geometry meshing, we got a number of around 20,000–50,000 elements (depending on the geometry size) with cell size ranging from 10^–4^–10^–6^ m^3^. After meshing, we then checked the skewness of cells. This step reassures us that there was no deformed mesh, which could affect the final result of our simulation. All of the skewness in our studying cases were kept under 0.85.

### Setting Up CFD Parameters

The first step of setting up CFD simulation was to load our meshed geometry into ANSYS Fluent. After loading, we defined blood density, viscosity, inlet velocity, and pressure of inlet and outlet. Finally, we selected our model for turbulence simulation. Trials were made previously to decide the best model for blood flow simulation. We compared all models in ANSYS Fluent with MRI results of blood flow and found that the k-omega SST model best describes blood turbulence. After selecting a model, we selected a calculation method. The method we used is the Green-Gauss Node-based method. The reason is that our geometry has tetrahedral cells and hexahedral cells. The Green-Gauss Node-based method prevents skewness error when calculating the results. Finally, we calculated our solution. Each residue in the calculation had reached the convergence criteria of 10^–5^, indicating that there were no calculation errors.

### Hemodynamic Analysis: CFD Simulations and Post-CFD Processing

As shown in Figure 1, a patient-specific 3D geometrical model of AVF venous lumen was built for each patient according to the specific shape and anastomotic angle of AVF on angiographic images. While the internal diameter of the radial artery (RA) was defined as 4 mm for all patients as previously adopted (Ene-Iordache et al., 2003; Ene-Iordache and Remuzzi, 2012), the diameter and shape of the anastomosis and the venous segment of each AVF were generated according to the patient-specific angiographic image. Five locations of AVF were defined, including the RA, the AV anastomotic site (Ana), AVF stenotic lesion site (Lesion), the proximal downstream site (PDS), and the distal downstream site (DDS). The RA site was the location, which was 20 mm upstream of the AV anastomotic site. The stenotic site was defined as the location with the minimal diameter of the AVF lumen. PDS was defined as the location, which was 20 mm immediately downstream of the stenotic lesion site, and DDS was 20 mm downstream of PDS.

Hemodynamic computational simulations of AVF were conducted in ANSYS Fluent 18.0 to obtain blood flow velocity in the lumen, and the corresponding WSS profiles were then calculated. The vessel wall was assumed to be rigid, immobile, and flow non-slip. Although the fistula veins are distensible to some extent (Corpataux et al., 2002), the rigid-wall assumption has been shown not to alter the flow field characteristics significantly (Perktold and Rappitsch, 1995). Flow at the inlet boundary condition (which was the inflow portion of the radial artery) and the two outlet boundary conditions (which were the AVF vein and the outflow portion of the radial artery) were prescribed as pulsatile and fully developed. For all patients, the cross-sectional average blood flow volume of the RA was set as 600 mL/min as previously reported for a mature AVF (Ene-Iordache et al., 2003); 400 and 800 mL/min were used for sensitivity analysis. The velocity for the outlet boundary conditions depend on the velocity for the inlet boundary condition, and the outlet flow volume was split proportionally by the cross-sectional area. The Reynolds numbers ranged from 516 to 1488 in our simulation domain, and thus, the laminar flow assumption was used (Zarins et al., 1983). Also, we assumed that blood is incompressible and Newtonian, and the blood density and viscosity were set as 1050 kg/m^3^ and 0.0035 N/m^2^, respectively, as previously reported (He et al., 2013).

After completing CFD simulations, we analyzed vectors of blood flow trajectories in the five locations of AVF. Quantitative characterization of the blood flow vector was analyzed by *OrientationJ*, an *ImageJ* plugin (National Institutes of Health, Bethesda, MD, United States) as previously reported (Rezakhaniha et al., 2012). In order to generate color-coded maps that depict blood flow trajectories (Figure 1F), *OrientationJ* raw output values were adjusted by assigning the angle between the blood flow trajectories and the axial direction of the AVF lumen ranged from −90 to 90°. For a blood flow trajectory parallel to the axial direction of the AVF, its angle of deviation is 0°, whereas for a blood flow trajectory perpendicular to the axial direction of the AVF, its angle of deviation is −90° or 90°. Each simulated blood flow’s angle of deviation was used to define its own trajectory vector (Figure 1F). We then summed up every vector to illustrate the distribution plot of blood flow vectors, as shown in Figure 1G. The degree of the most abundant vectors was denoted as the blood flow disturbance.

### Statistical Analysis

Chi-square analysis or Fisher’s exact test was used for comparison of categorical variables as appropriate. Continuous variables were compared by Student’s *t*-test, paired *t*-test, or Pearson’s correlation as appropriate. Pearson’s correlation coefficient was used to evaluate bivariate relationships between the AV anastomotic angle and hemodynamic parameters. Receiver operating characteristic (ROC) curve analyses were performed to determine the best cut-off values, at which a sum of the sensitivity/specificity pair is the maximum, for predicting an AV angle that discriminates high and low disturbed flow. Also, two additional independent CFD analyses were performed as sensitivity analyses by setting the simulated RA blood flow as 400 and 800 mL/min, respectively. SPSS version 18.0 for Windows (SPSS Inc., Chicago, Illinois, United States) was used for all statistical analyses. All probabilities were two-tailed, and a *p*-value of less than 0.05 was considered to be statistically significant.

## Results

### Demographic Characteristics of Study Participants

Table 1 shows the demographic characteristics of the 27 patients at the time of the PTA procedure. The mean age was 71 years, 56% of patients were male, and the mean duration on dialysis was 14.3 months. A total of 20 patients (74%) had diabetes mellitus, 23 patients (85%) had hypertension, 14 patients (52%) had coronary artery disease, 11 patients (41%) had congestive heart failure, three patients (11%) had a history of stroke, and six patients (22%) had malignancy. The AV anastomotic angle was 51.3 ± 22.5°, and the distance between anastomosis and stenotic lesion site was 21.9 ± 11.3 mm. Besides, a wider AV angle is associated with more severe stenosis at the lesion site (Figure 1B and Table 1).

**TABLE 1:** Demographic, angiographic, and hemodynamic characteristics of study participants.

	AV anastomotic angle		Total	*p*-value
	<46.5°	≧46.5°		
Patient number (*n*)	16	11	27	
Age (year)	71.0 ± 10.4	71.2 ± 11.7	71.1 ± 10.7	0.972
Male gender (*n*; %)	11; 68.8	4; 36.6	15; 55.6	0.130
Hemodialysis vintage (month)	14.6 ± 10.9	14.0 ± 14.4	14.3 ± 12.1	0.902
Time interval between AVF creation and angiography (month)	13.8 ± 9.8	13.9 ± 12.2	13.8 ± 10.6	0.970
**Underlying renal diseases**				
Diabetic nephropathy (*n*; %)	7; 43.8	4; 36.4	11; 40.7	1.000
Hypertensive nephropathy (*n*; %)	5; 31.3	1; 9.1	6; 22.2	0.350
Immune-mediated glomerulonephritis (*n*; %)	1; 6.3	2; 18.2	3; 11.1	0.549
Chronic tubulointerstitial nephritis (*n*; %)	1; 6.3	3; 27.3	4; 14.8	0.273
Others (*n*; %)	2; 12.5	1; 9.1	3; 11.1	1.000
**Comorbidities**				
Diabetes mellitus (*n*; %)	12; 75.0	8; 72.7	20; 74.1	1.000
Hypertension (*n*; %)	14; 87.5	9; 81.8	23; 85.2	1.000
Coronary artery disease (*n*; %)	9; 56.3	5; 45.5	14; 51.9	0.581
Congestive heart failure (*n*; %)	9; 56.3	2; 18.2	11; 40.7	0.109
Prior stroke (*n*; %)	2; 12.5	1; 9.1	3; 11.1	1.000
Malignancy (*n*; %)	2; 12.5	4; 36.4	6; 22.2	0.187
**Angiographic parameters**				
AV anastomotic angle (°)	37.3 ± 7.2	71.8 ± 21.5	51.3 ± 22.5	<0.001*
Anastomosis-to-lesion distance (mm)	25.0 ± 17.4	17.4 ± 10.1	21.9 ± 11.3	0.089
Stenotic ratio of the lesion (%)	54.4 ± 7.0	63.7 ± 12.4	58.2 ± 10.4	0.019
**Hemodynamic parameters at the stenotic lesion site**				
Blood flow velocity (cm/sec)	68.6 ± 16.7	94.5 ± 16.4	79.1 ± 20.8	0.001*
Reynolds number	823.0 ± 200.1	1134.0 ± 196.9	949.7 ± 249.5	0.001*
Wall shear stress (N/m^2^)	6.0 ± 4.5	9.1 ± 4.0	7.3 ± 4.5	0.074
Blood flow disturbance (°)	15.5 ± 13.4	52.8 ± 25.7	30.7 ± 26.6	<0.001*

Data were presented as mean ± SD or percentage as appropriate. P-values denote the significance of the difference between patients with <46.5 and >46.5° anastomosis angle. AV, arteriovenous; AVF, arteriovenous fistula; CFD, computational fluid dynamics. *p < 0.05.

### Hemodynamic Parameters at Five Different Locations of AVF

**FIGURE 2:**
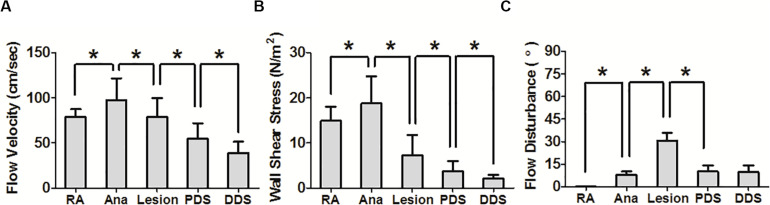
Computational fluid dynamics analysis at five different locations of the arteriovenous fistula. **(A,B)** The flow velocity and wall shear stress were the highest at the anastomosis. **(C)** The disturbed flow was the highest at the stenotic lesion site. RA, radial artery; Ana, anastomosis; PDS, proximal downstream site; DDS, distal downstream site. *p < 0.05.

As shown in Figure 2, flow velocity was the highest at the anastomotic site (mean ± SD = 97.5 ± 24.1 cm/sec), followed by the stenotic lesion site (79.1 ± 20.8 cm/sec), RA (79.0 ± 8.5 cm/sec), PDS (54.6 ± 17.4 cm/sec), and DDS (39.1 ± 12.5 cm/sec). Next, WSS was the highest at the anastomotic site (18.8 ± 6.0 N/m^2^), followed by RA (14.9 ± 3.1 N/m^2^), the stenotic lesion site (7.3 ± 4.5 N/m^2^), PDS (3.8 ± 2.2 N/m^2^), and DDS (2.1 ± 0.8 N/m^2^). In contrast, the disturbed flow was the highest at the stenotic location (30.7 ± 26.6°), followed by PDS (10.1 ± 19.9°), DDS (9.6 ± 22.8°), the anastomotic site (8.0 ± 11.0°), and RA (0.2 ± 0.6°).

### The Relationship Between the AV Anastomotic Angle and Hemodynamic Parameters at the Stenotic Lesion Site

**FIGURE 3:**
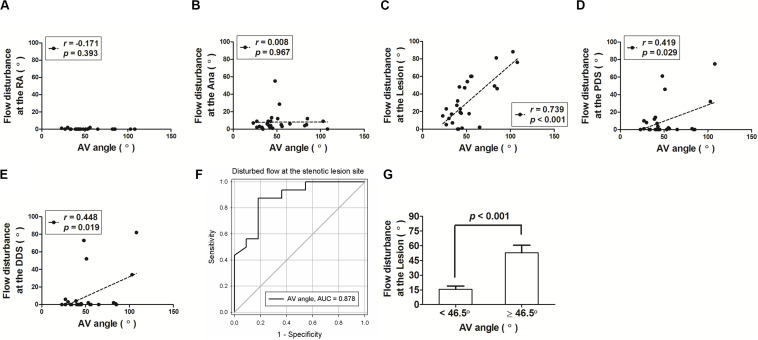
The arteriovenous anastomotic angle (AV angle) significantly correlated with the blood flow disturbance of the arteriovenous fistula (AVF), particularly at the stenotic lesion site. **(A–E)** Correlation dot plots between AV angle and flow disturbance at five different locations of the AVF. RA, radial artery; Ana, anastomosis; PDS, proximal downstream site; DDS, distal downstream site. **(F)** The receiver operating characteristic (ROC) curve analysis revealed that the AV angle determines the lesion’s flow disturbance with a high area under the curve value of 0.878. **(G)** The ROC analysis identified a cut-off value of AV angle as 46.5°, above or below which the AVF flow disturbance was significantly different.

Next, we examined the relationship between the AV anastomotic angle and hemodynamic parameters at the five locations, with a focus on the stenotic lesion site. We found that the AV anastomotic angle was the most significantly correlated with the disturbed flow at the stenotic lesion site (*r* = 0.739; *p* < 0.001), followed by DDS (*r* = 0.448; *p* = 0.019) and PDS (*r* = 0.390; *p* = 0.029), but was not associated at RA (*r* = -0.171; *p* = 0.393) and the anastomotic site (*r* = 0.008; *p* = 0.967) (Figures 3A–E). Additionally, at the stenotic lesion site, the AV anastomotic angle was also correlated with flow velocity (*r* = 0.563; *p* = 0.002) but not with WSS (*r* = 0.251; *p* = 0.208).

### ROC Curve Analysis to Identify the Optimal AV Anastomotic Angle With a Lower Disturbed Flow at the Stenotic Lesion Site of AVF

To examine the optimal anastomotic angle of AVF for lower flow disturbance, we did a ROC analysis, which revealed a high area under the curve value of 0.878 (Figure 3F). Next, we summed sensitivity/specificity pairs to determine the cut-off value of the AV anastomotic angle, which turns out to be 46.5°. We found that the AVF disturbed flow was significantly higher when the AV anastomotic angle was above 46.5° when compared to those with the angle below 46.5° (Figure 3G and Table 2).

**TABLE 2:** Arteriovenous fistula (AVF) blood flow disturbance, as denoted by the degrees of the most abundant vector, between AV angle <46.5° or ≥46.5° with AVF blood flow at 600 mL/min.

	AV anastomotic angle		*p*-value
	<46.5°	≥46.5°	
Patient number (*n*)	16	11	
Radial artery	0.2 ± 0.5	0.3 ± 0.6	0.714
AV anastomotic site	4.8 ± 3.7	12.7 ± 16.0	0.137
Stenotic lesion site	15.5 ± 13.4	52.8 ± 25.7	<0.001*
Proximal downstream site	3.5 ± 5.0	19.8 ± 28.6	0.089
Distal downstream site	0.9 ± 1.8	22.3 ± 32.3	0.052

* p < 0.05. AV, arteriovenous.

### Sensitivity Analysis

Finally, we conducted two additional independent CFD analyses, by using simulated RA blood flow of 400 and 800 mL/min, to validate our findings. Their simulated results were similar to those of 600 mL/min for hemodynamic parameters (Table 3 and Supplementary Table 1) and ROC curve analysis (Supplementary Figure 1). On the other hand, we aimed to predict the next stenotic location if an angioplasty was not performed during the angiographic procedure, and the original lesion remained stenotic. We further built a 3D vascular model based on the angiographic image before the angioplasty of the stenotic lesion (Figure 1A). As shown in Supplementary Figure 2, by using the visualization of velocity vector distribution at different locations of the AVF, the flow disturbance was more profound in the PDS than that of DDS, which might explain the stenotic lesion progression in the clinical scenario.

**TABLE 3:** The correlation between arteriovenous fistula (AVF) blood flow disturbance and the AV anastomotic angle at different AVF blood flow settings.

AVF blood flow (mL/min)		Stenotic lesion site	Proximal downstream site	Distal downstream site
600	Pearson correlation	0.739	0.419	0.448
	*p*-value	<0.001*	0.029*	0.019*
400	Pearson correlation	0.694	0.420	0.437
	*p*-value	<0.001*	0.029*	0.023*
800	Pearson correlation	0.615	0.459	0.465
	*p*-value	0.001*	0.016*	0.014*

* p < 0.05. AVF, arteriovenous fistula.

## Discussion

### The Optimal AV Anastomotic Angle

The AV anastomotic angle has been shown to affect disturbed flow generation based on theoretical CFD simulations using standardized and simplified geometry (Ene-Iordache and Remuzzi, 2012; Ene-Iordache et al., 2013). Our study created individualized AVF geometry from angiographic images, presenting a novel approach to integrate the patient-specific AVF contour information into the CFD simulations. The angiographic images also had the advantages of providing the patient-specific outcomes, i.e., the location of the AVF stenotic lesion in each patient. We discovered that the AVF anastomotic angle was associated and hence may determine the flow disturbance at the location of the stenotic lesion. Based on angiographic images with stenosis, the cut-off value of the AV angle of our study was 46.5°, below which the lesion’s flow disturbance is reduced. This value is larger than the result of a theoretical CFD study, showing that the optimal angle should be less than 30° (Ene-Iordache et al., 2013). However, it is important to note that there were still many newly created AVF with an angle wider than 45° nowadays, including some AVFs in our study. Our results not only substantiate the importance of adopting an optimal AV anastomotic angle but also provide patient-specific evidence for the pathogenetic role of disturbed flow derived from a wide angle.

### Blood Flow Disturbance and Intimal Hyperplasia

The characteristics of fluidic flow include laminar and disturbed flow, and fluid shear stress increases as the flow become disturbed (Chien, 2007; Chiu and Chien, 2011). Cell experiments indicated that laminar flow increases endothelial cell survival, whereas disturbed flow leads to endothelial dysfunction (Chien, 2008a, b), followed by smooth muscle cell proliferation and intimal migration (Paszkowiak and Dardik, 2003; Marie et al., 2014). Our results demonstrated that among the five different locations of AVF, the highest disturbed flow occurs at the stenotic lesion site. By using standardized and simplified AVF geometry, two previous CFD studies found that disturbed flow locates at the stenotic site (Ene-Iordache and Remuzzi, 2012), and the anastomotic angle affects disturbed flow (Ene-Iordache et al., 2013). Although our finding is consistent with these previous studies, our analysis is advancement by using real-world, patient-specific, angiographic image-derived AVF geometry. In addition, our research revealed an interconnected link among the anastomotic angle, the flow disturbance, and the stenotic site; specifically, when compared to the other locations in the AVF, blood flow disturbance at the stenotic site shows the strongest correlation with the AV anastomotic angle. The reconstructed patient-specific geometrical model has become a powerful tool to investigate disturbed hemodynamics and stenosis in cardiovascular diseases (Salman et al., 2019; Wang et al., 2019).

### Etiologic Mechanisms of Juxta-Anastomotic Stenosis

The 1-year primary patency rate of AVF has not changed over the decades, ranging from 34 to 56% (Leapman et al., 1996; Hodges et al., 1997; Turmel-Rodrigues et al., 2000; Gibson et al., 2001; Clark et al., 2007; Huijbregts et al., 2008; Li et al., 2008). Stenosis is a critical factor of AVF failure. As the surgical procedure is a critical factor responsible for AVF stenosis (Roy-Chaudhury et al., 2006; Lee and Roy-Chaudhury, 2009), our results emphasize the contribution of the AV angle to disturbed flow generation in AVF. On the other hand, the etiologic mechanisms of juxta-anastomotic stenosis are multi-factorial, such as injury to the vasa venorum during mobilization of the vein (Osgood et al., 2014; Yang et al., 2014). However, since only patients with a healthy ulnar artery are eligible for the RADAR technique (i.e., an arterial end to venous side anastomosis), the standard technique (i.e., an arterial side to venous end anastomosis) is still widely used nowadays. Under such circumstances, our findings could provide the surgeons with a useful reference to reduce the stenosis-inducing disturbed flow formation.

A previous CFD study denoted that an anastomosis angle of 30° is a preferred choice as compared to 45°, 60°, and 90° (Ene-Iordache et al., 2013). On the other hand, in a patient data-based study, though the authors did not identify a cut-off value for an optimal anastomotic angle in their research, they reported that the anastomotic angle was 42 ± 11° in stenosis non-progressors as compared to 49 ± 13° in progressors (Sivanesan et al., 1999a). In our study cohort, only three patients (11%) had an AVF angle of less than 30°. This surgical characteristic reflects the fact that in clinical practice, it might be challenging to adopt an extremely sharp anastomotic angle during surgery when taken the patients’ vessel anatomy and technical limits into consideration. Sadaghianloo et al. (2015) also suggested that surgeons should avoid an anastomotic angle of less than 30° when creating radio-cephalic AVFs in a prospective study, while the VasQ (Laminate Medical Technologies, Israel) external metallic device creates a 60° anastomotic angle between the radial artery and cephalic vein to optimize hemodynamic conditions (Tordoir et al., 2018). Our study provides additional evidence showing that an anastomotic angle of more than 46.5° might lead to disturbed flow generation and more severe stenosis, which supports future trials with the devices that are available to determine the anastomotic angle, such as the VasQ implant. In our study, the anastomosis angle is more than 46.5° in almost half of our patients (12 out of 27), meaning that there is still room for surgical improvement.

### Limitations

There were some limitations in our study. Firstly, our 3D geometrical models of AVF lumen were derived from the 2D angiographic images at the time of angioplasty. Nonetheless, we selected perpendicularly projected images from a series of images of each patient. Future computational studies using 3D vascular models built by magnetic resonance angiography may be a promising method to validate our findings (He et al., 2013; Bozzetto et al., 2018). Secondly, the definition of an internal diameter of the radial artery is defined based on the literature (Ene-Iordache et al., 2003; Ene-Iordache and Remuzzi, 2012). Thirdly, we did not use the disturbed flow categorized in regions of interest of wall surface by hemodynamic parameters (e.g., oscillatory shear index, transWSS, relative residence time), and we did not adopt patient-specific inlet blood flow rate and blood viscosity, which deserve further investigations. Last, we did not include angiographic images without stenosis because, in clinical practice, only AVFs that are suspected of stenosis will receive angiography. However, we did demonstrate the relationship between AV angle and the stenotic severity.

## Conclusion

We integrated the patient-specific AVF geometry into our CFD simulations by using real-world angiography images, instead of using a monotonous and idealized shape. Compared to other upstream and downstream sites of a radio-cephalic AVF, the disturbed flow maximizes at the location of the stenotic lesion. Furthermore, at the stenotic lesion site of AVF, among the three hemodynamic parameters, flow disturbance outweighed the other two parameters in terms of correlation to the AV anastomotic angle. Our integrative findings provide a reference range of the anastomotic angle that might reduce disturbed flow generation.

## Data Availability Statement

All datasets generated for this study are included in the article/Supplementary Material.

## Ethics Statement

The studies involving human participants were reviewed and approved by the Institutional Review Board of the Taipei Veterans General Hospital. Written informed consent for participation was not required for this study in accordance with the national legislation and the institutional requirements.

## Author Contributions

C-YY, M-CL, Y-TS, AC, Y-HL, OL, SC, and D-CT: study design and data interpretation. C-YY and C-HW: data collection. C-YY, M-CL, C-WL, W-JL, C-JL, J-MT, Y-YN, and Y-PL: data analysis. C-YY and M-CL: drafting the manuscript. C-YY, Y-TS, and D-CT: revising the manuscript content. All authors contributed to the article and approved the submitted version.

## Conflict of Interest

The authors declare that the research was conducted in the absence of any commercial or financial relationships that could be construed as a potential conflict of interest.
