# Supplementary material for: The Anastomotic Angle of Hemodialysis Arteriovenous Fistula Is Associated With Flow Disturbance at the Venous Stenosis Location on Angiography
Source: Front Bioeng Biotechnol. 2020 Jul 23;8:846. doi: 10.3389/fbioe.2020.00846 (PMC7390971; doi:10.3389/fbioe.2020.00846)
Supplement: Supplementary file 1 [file Table_1.docx]

Supplementary Material

## Supplementary Figures


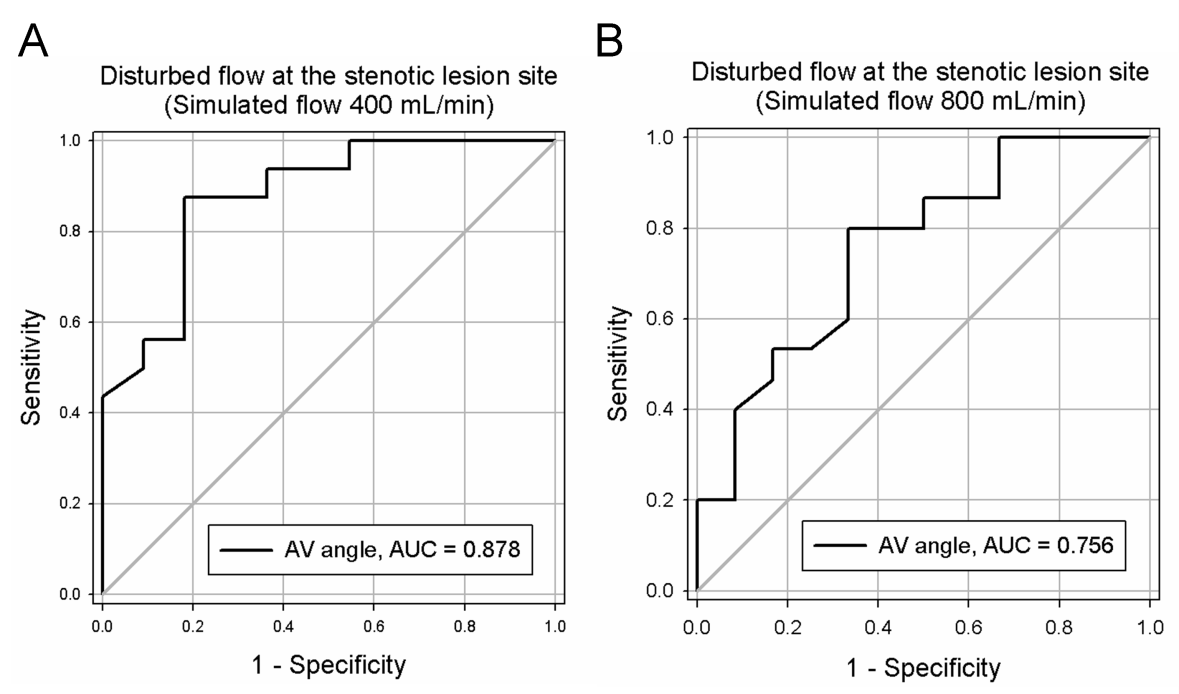


**Supplementary Figure 1.** The receiver operating characteristic curve analysis revealed that the arteriovenous anastomotic angle (AV angle) determines the lesion’s flow disturbance with an area under the curve (AUC) value of 0.878 and 0.756, while the simulated blood flow was set as 400 mL/min (**A**) and 800 mL/min (**B**), respectively.

**
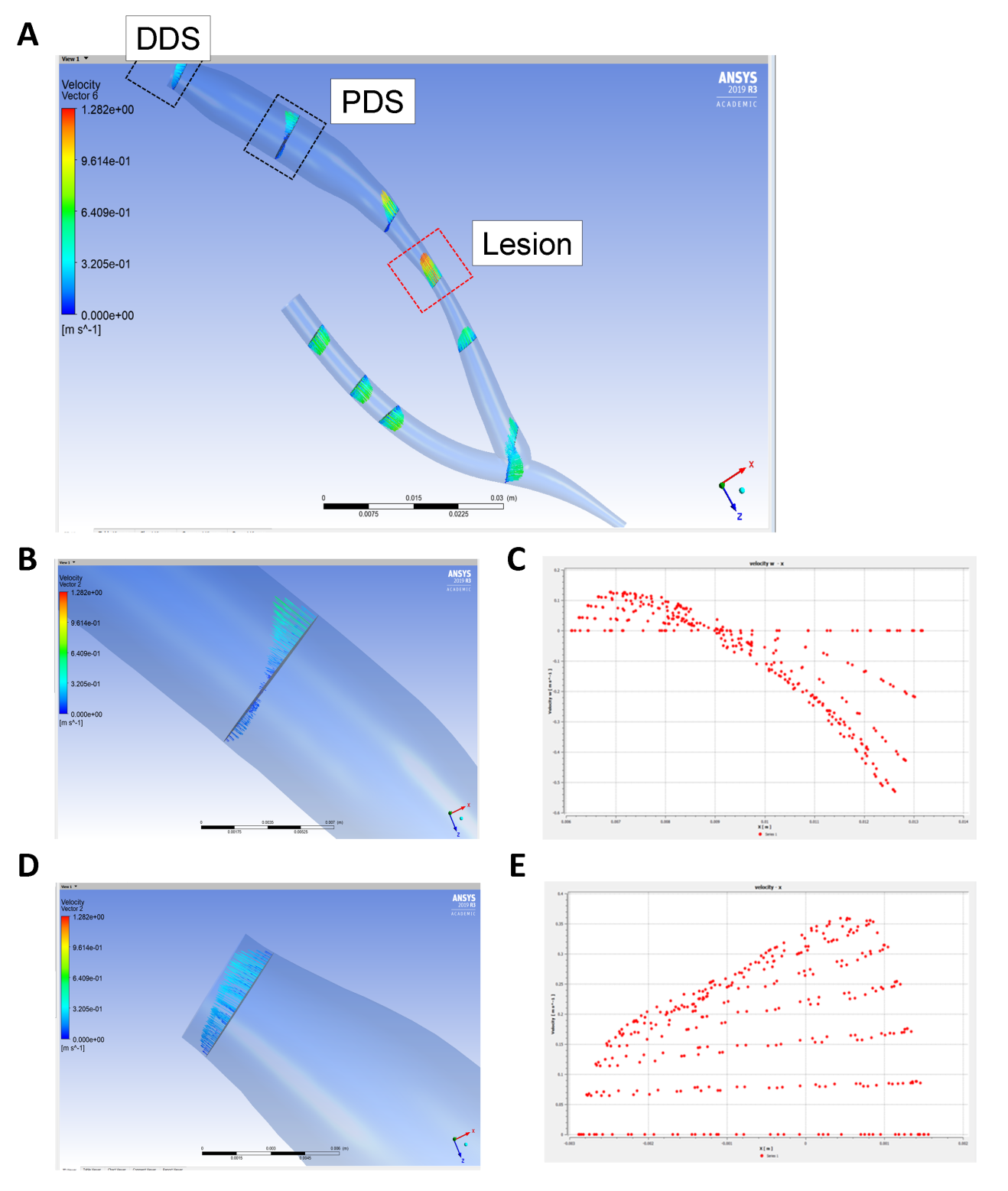
**

**Supplementary Figure 2.** (**A**) The representative visualization of velocity vector distribution by computational fluid dynamics analysis at different locations of the arteriovenous fistula, which was built from the angiographic image performed before percutaneous transluminal angioplasty for the juxta-anastomotic stenotic lesion. (**B-C**) The flow velocity and corresponding velocity scatter plot at the proximal downstream site, showing a higher disturbed flow than the distal downstream site. (**D-E**) The flow velocity and corresponding velocity scatter plot at the distal downstream site. Abbreviations: PDS, proximal downstream site, DDS, distal downstream site.

## Supplementary Tables

| **Supplementary Table 1.** Arteriovenous fistula (AVF) blood flow turbulence, as denoted by the degrees of the peak vector, between AV angle＜46.5° or ≧46.5° with AVF blood flow at 400 mL/min and 800 mL/min, respectively (*n* = 27). | | | | | | | |
| --- | --- | --- | --- | --- | --- | --- | --- |
| AVF blood flow | 400 mL/min | | |  | 800 mL/min | | |
|  | AV anastomotic angle | | *p*-Value |  | AV anastomotic angle | | *p*-Value |
|  | ＜46.5° | ≧46.5° |  |  | ＜46.5° | ≧46.5° |  |
| Patient number (*n*) | 16 | 11 |  |  | 16 | 11 |  |
| Radial artery | 0.4 ± 0.8 | 0.0 ± 0.0 | 0.048* |  | 0.0 ± 0.0 | 0.2 ± 0.6 | 0.341 |
| AV anastomotic site | 12.7 ± 10.6 | 9.5 ± 8.9 | 0.406 |  | 7.1 ± 6.3 | 4.9 ± 2.5 | 0.293 |
| Stenotic lesion site | 16.6 ± 14.8 | 50.2 ± 22.7 | < 0.001* |  | 16.0 ± 14.1 | 44.9 ± 25.7 | 0.004* |
| Proximal downstream site | 3.7 ± 6.1 | 23.8 ± 27.3 | 0.035* |  | 3.2 ± 4.9 | 23.3 ± 27.9 | 0.039* |
| Distal downstream site | 0.6 ± 1.6 | 21.0 ± 30.3 | 0.049* |  | 0.5 ± 1.3 | 22.7 ± 33.0 | 0.050 |
| **p* < 0.05. Abbreviations: AVF, arteriovenous fistula; AV, arteriovenous. | | | | | | | |
